# Supplementary material for: Declining harbour seal abundance in a previously recovering meta-population
Source: PLoS One. 2025 Jun 30;20(6):e0326933. doi: 10.1371/journal.pone.0326933 (PMC12208499; doi:10.1371/journal.pone.0326933)
Supplement: S1 Fig — (PDF) [file pone.0326933.s001.pdf]

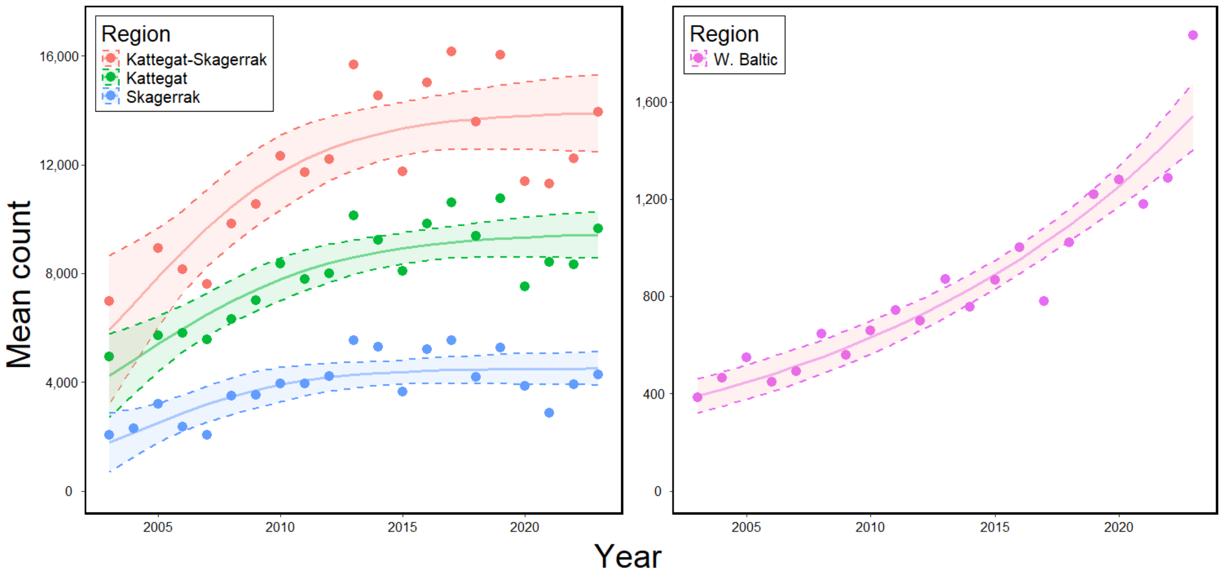

S1 Fig. Logistic growth models (solid lines) were the best fit for moult count data (points) when the Kattegat-Skagerrak was considered as a whole (pink), or when broken down into the Kattegat (green) and the Skagerrak (blue). The exponential growth model was found to be the best fit for the Western Baltic (W. Baltic, purple). Dashed lines represent 95 % confidence intervals of estimates. See S2 Table for details on model selection and parameter values.
