# Supplementary material for: Declining harbour seal abundance in a previously recovering meta-population
Source: PLoS One. 2025 Jun 30;20(6):e0326933. doi: 10.1371/journal.pone.0326933 (PMC12208499; doi:10.1371/journal.pone.0326933)
Supplement: S2 Fig — (PDF) [file pone.0326933.s002.pdf]

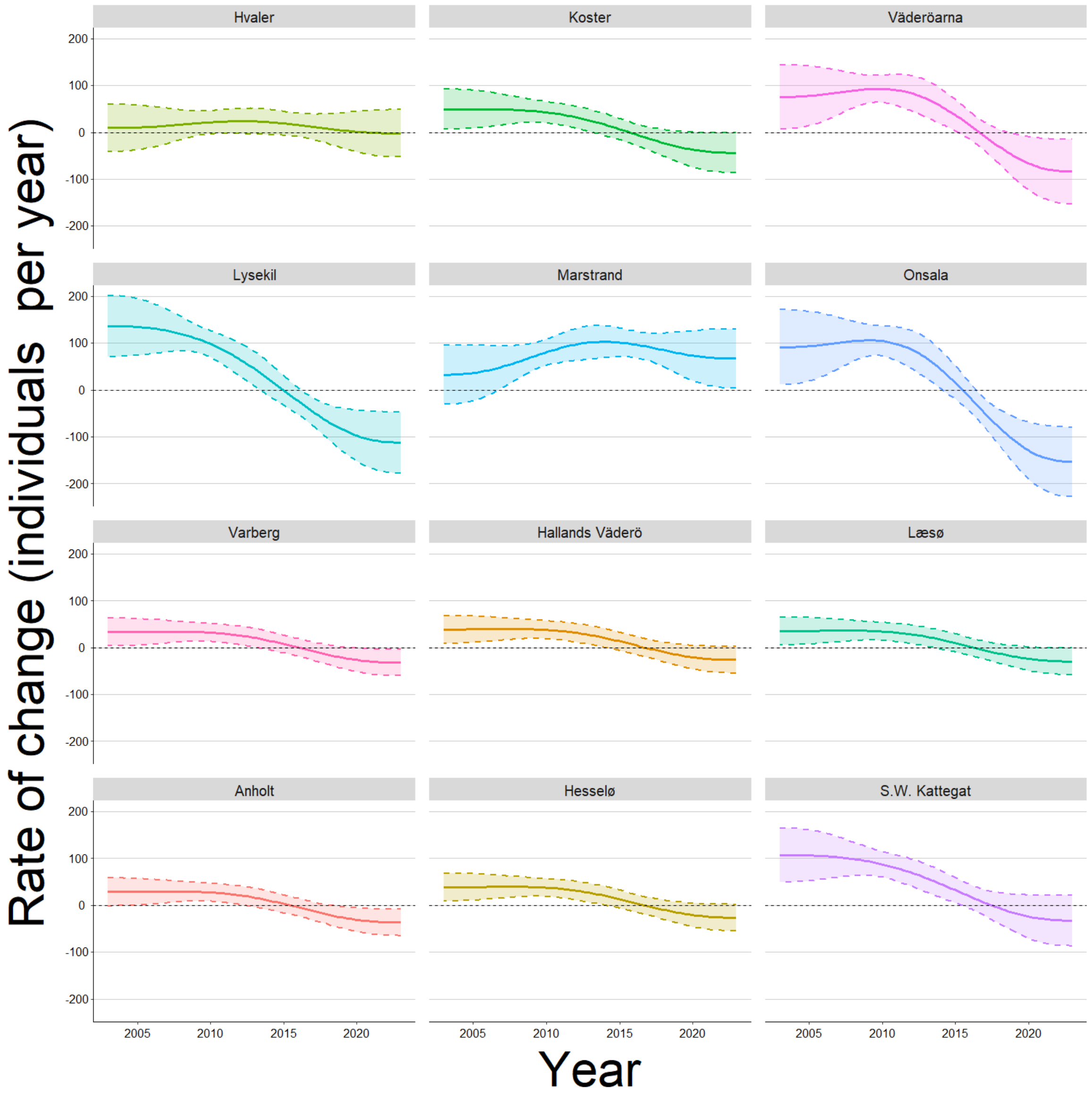

S2 Fig. Rates of change for subregions were estimated based on the first derivative of a non-parametric generalised additive model (GAM) fit to mean counts from moult surveys (points). Dashed lines represent 95 % confidence intervals of estimates. Horizontal black dashed line is placed at zero, representing no growth. See S4 Table for details on model fitting.
