# Supplementary material for: Declining harbour seal abundance in a previously recovering meta-population
Source: PLoS One. 2025 Jun 30;20(6):e0326933. doi: 10.1371/journal.pone.0326933 (PMC12208499; doi:10.1371/journal.pone.0326933)
Supplement: S1 Table — (PDF) [file pone.0326933.s003.pdf]

S1 Table. Summary of counts for colonies in Norway surveyed in 2016 and 2022 only.

Year	Colony	Count 1	Count 2	Count 3	Mean
2016	1. Farsund	34	NA	NA	NA
	2. Tvedestrand	28	26	30	28
	3. Kragerø	90	175	142	135.67
	4. Færder	155	157	292	201.33
	Total				365
2022	1. Farsund	29	38	46	37.67
	2. Tvedestrand	67	64	68	66.33
	3. Kragerø	179	218	184	193.67
	4. Færder	218	599	263	360
	Total				657.67
