# Supplementary material for: Declining harbour seal abundance in a previously recovering meta-population
Source: PLoS One. 2025 Jun 30;20(6):e0326933. doi: 10.1371/journal.pone.0326933 (PMC12208499; doi:10.1371/journal.pone.0326933)
Supplement: S2 Table — (PDF) [file pone.0326933.s004.pdf]

S2 Table. The outcomes of parametric modelling. Mean counts for moult surveys were summarised and fit with logistic and exponential models for the combined Kattegat-Skagerrak and for each of the subregions (the Kattegat, the Skagerrak, and the S.W. Baltic). RSE = Residual standard error. DF = degrees of freedom. AIC = Akaike information criterion. Parm. = parameter. SE = standard error.

**Region**	**Mod.**	**RSE (DF)**	**AIC**	**Parm. Est. ± SE**	***t*-value**	**p-value**
Kattegat-Skagerrak	Log.	1,708 (17)	359.23	K = 13,965 ± 772	18.09	< 0.001
				N_0 = 5,930 ± 1,290	4.60	< 0.001
				μ = 0.28 ± 0.10	2.67	0.016
	Exp.	2,162 (18)	367.81	N_0 = 9,285 ± 867	10.71	< 0.001
				r = 0.02 ± 0.01	3.38	0.003
Kattegat	Log.	977 (17)	336.89	9,509 ± 510	18.66	< 0.001
				4,242 ± 726	5.84	< 0.001
				0.25 ± 0.08	2.95	0.009
	Exp.	1,246 (18)	345.77	6,159 ± 496	12.42	< 0.001
				0.03 ± 0.01	4.16	< 0.001
Skagerrak	Log.	787 (18)	344.43	4,517 ± 305	14.81	< 0.001
				1,778 ± 514	3.46	0.003
				0.33 ± 0.15	2.16	0.045
	Exp.	962 (19)	352.00	3,013 ± 361	8.36	< 0.001
				0.02 ± 0.01	2.58	0.018
S.W. Baltic	Exp.	119 (19)	264.51	391 ± 34	11.47	< 0.001
				0.07 ± 0.01	12.03	< 0.001
