# Supplementary material for: Declining harbour seal abundance in a previously recovering meta-population
Source: PLoS One. 2025 Jun 30;20(6):e0326933. doi: 10.1371/journal.pone.0326933 (PMC12208499; doi:10.1371/journal.pone.0326933)
Supplement: S3 Table — (PDF) [file pone.0326933.s005.pdf]

S3 Table. Estimates of count (Est. count) based on Generalised Additive Models (GAMs) for the Kattegat-Skagerrak and for each region. Estimates and associated 95 % confidence intervals ($CI_{95\%}$) are shown for the maximum estimated count (Max.) and the most recent survey year (2023).

Region	Year	Estimated count ($CI_{95\%}$)
Kattegat-Skagerrak	2017 (Max.)	14,189 (14,739, 13,638)
	2023	12,507 (13,456, 11,558)
Kattegat	2017 (Max.)	9,540 (9,785, 9,295)
	2023	8,640 (8,225, 9,054)
Skagerrak	2016 (Max.)	4,767 (5,000, 5,435)
	2023	3,710 (3,309, 4,111)
S.W. Baltic	2023 (Max.)	1,388 (1,657, 1,118)
