# Supplementary material for: Declining harbour seal abundance in a previously recovering meta-population
Source: PLoS One. 2025 Jun 30;20(6):e0326933. doi: 10.1371/journal.pone.0326933 (PMC12208499; doi:10.1371/journal.pone.0326933)
Supplement: S4 Table — (PDF) [file pone.0326933.s006.pdf]

S4 Table. Outcomes of GAM fitting for the Kattegat-Skagerrak including a main effect and interaction of subregion. An asterisk (*) indicates an interaction. SE = standard error. EDF = effective degrees of freedom. Ref. DF = reference degrees of freedom.

**Main effects**				
	**Intercept**	**SE**	***t*-value**	***p*-value**
Hvaler	275	49	5.67	< 0.001
Koster	1278	69	14.6	< 0.001
Väderöarna	1108	69	12.12	< 0.001
Lysekil	1227	69	13.86	< 0.001
Marstrand	963	69	10.02	< 0.001
Onsala	1706	70	20.58	< 0.001
Varberg	772	70	7.14	< 0.001
Hallands Vaderö	645	70	5.33	< 0.001
Læsø	918	70	9.25	< 0.001
Anholt	713	70	6.3	< 0.001

Hesselø	1106	70	11.95	< 0.001
S.W. Kattegat	1169	70	12.87	< 0.001
**Approximate significance of smooth terms**				
	**EDF**	**Ref. DF**	***f*-value**	***p*-value**
Year	2.25	2.58	9.68	< 0.001
Year*Hvaler	1.65	2.02	1.01	0.368
Year*Koster	1.45	1.78	0.59	0.584
Year*Väderöarna	2.36	2.69	4.43	0.008
Year*Lysekil	2.22	2.58	8.34	< 0.001
Year*Marstrand	2.15	2.53	19.04	< 0.001
Year*Onsala	2.51	2.79	5.57	< 0.001
Year*Varberg	0.92	0.92	0.33	0.584
Year*Hallands Väderö	0.92	0.92	0.09	0.772
Year*Læsø	0.92	0.92	0.1	0.762

Year*Anholt	0.92	0.92	1.15	0.304
Year*Hesselø	0.92	0.92	0.001	0.983
Year*S.W. Kattegat	1.8	2.19	5.91	0.002
