# Supplementary material for: Declining harbour seal abundance in a previously recovering meta-population
Source: PLoS One. 2025 Jun 30;20(6):e0326933. doi: 10.1371/journal.pone.0326933 (PMC12208499; doi:10.1371/journal.pone.0326933)
Supplement: Attachment — Submitted filename: PONE-D-25-11452_reviewer.pdf [file pone.0326933.s008.pdf]

PLOS ONE

Declining harbour seal abundance in the Kattegat-Skagerrak and Danish Straits

--Manuscript Draft--

**Manuscript Number:**	PONE-D-25-11452
**Article Type:**	Research Article
**Full Title:**	Declining harbour seal abundance in the Kattegat-Skagerrak and Danish Straits
**Short Title:**	Declining harbour seal abundance
**Corresponding Author:**	Daire Carroll, PhD<br>University of Gothenburg: Goteborgs Universitet<br>Gothenburg, SWEDEN
**Keywords:**	Harbour Seal; Phoca vitulina; population decline; environmental monitoring; population biology; environmental degradation
**Abstract:**	We present evidence that the abundance of harbour seals in the Kattegat-Skagerrak is in decline. Until recently, the Kattegat-Skagerrak harbour seal population has grown exponentially as it has recovered from historic over-hunting and two mass mortality events. This has provided an important case study for understanding the influence of environmental factors on population growth. Over recent years, deteriorating average body condition and reduced pup counts in certain colonies have indicated that the population is under stress. At the same time, there has been an increase in hunting in the region which may compound existing environmental stressors. To determine trends in harbour seal abundance, we compile aerial survey data for the Kattegat-Skagerrak and Danish Straits between the years 2003 and 2023. With parametric modelling, we find that logistic growth models are the best fit for aerial survey data from the Kattegat and Skagerrak, while an exponential growth model is the best fit for the smaller South-Western Baltic population. Across spatial scales ranging from sub-regions, representing individual colonies, up to the combined Kattegat-Skagerrak, we determine trends by fitting non-parametric generalised additive models (GAMs) to aerial survey data. In the Kattegat-Skagerrak, we find that rate of change has declined to zero or below zero in eleven out of twelve sub-regions, with an estimated annual decline of -408 individuals (SE = 242, CI95% = [67, -882]) for the combined Kattegat-Skagerrak in 2023. This represents approximately 7% of the mean count that year. Rate of change remained positive in the South-Western Baltic throughout the survey period at an estimated annual increase of 54 individuals (SE = 23, CI95% = [99, 9]). Probable causes of declining counts in the Kattegat-Skagerrak are a combination of environmental degradation causing regionally lowered birth rates, changing haul-out behaviour, and shifts in seal distribution to less densely populated areas.
**Order of Authors:**	Daire Carroll, PhD
	Markus P. Ahola
	Anja M. Carlsson
	Anders Galatius
	Kjell T. Nilssen
	Tero Härkönen
	Karin C. Harding
**Additional Information:**	
**Question**	**Response**
**Financial Disclosure**<br><br>Enter a financial disclosure statement that describes the sources of funding for the work included in this submission. Review	Yes

the submission guidelines for detailed requirements. View published research articles from *PLOS ONE* for specific examples.<br><br>This statement is required for submission and **will appear in the published article** if the submission is accepted. Please make sure it is accurate.<br><br>**Funded studies**<br>Enter a statement with the following details:<br>• Initials of the authors who received each award<br>• Grant numbers awarded to each author<br>• The full name of each funder<br>• URL of each funder website<br>• Did the sponsors or funders play any role in the study design, data collection and analysis, decision to publish, or preparation of the manuscript?<br><br>Did you receive funding for this work?	
Please add funding details.<br>as follow-up to "**Financial Disclosure**<br><br>Enter a financial disclosure statement that describes the sources of funding for the work included in this submission. Review the submission guidelines for detailed requirements. View published research articles from *PLOS ONE* for specific examples.<br><br>This statement is required for submission and **will appear in the published article** if the submission is accepted. Please make sure it is accurate.	Swedish surveys were funded by the Swedish Environmental Protection Agency, and the Swedish Agency for Marine and Water Management (no specific grant codes avalible). Danish surveys were funded by the Danish Environmental Protection Agency (no specific grant codes avalible). DC was funded by the Wild Animal Initiative Fellowship, grant numbers F-2023-00005 and the Swedish Environmental Protection Agency through the Environmental research fund and the Swedish Research Council Formas, grant number (2024-00147). KCH was supported by the European Union (HORIZON-CL6-2023-BIODIV-01-101135307).

**Funded studies**<br>Enter a statement with the following details:<br>• Initials of the authors who received each award<br>• Grant numbers awarded to each author<br>• The full name of each funder<br>• URL of each funder website<br>• Did the sponsors or funders play any role in the study design, data collection and analysis, decision to publish, or preparation of the manuscript?<br><br>Did you receive funding for this work?"	
Please select the country of your main research funder (please select carefully as in some cases this is used in fee calculation).<br>as follow-up to "**Financial Disclosure**<br><br>Enter a financial disclosure statement that describes the sources of funding for the work included in this submission. Review the submission guidelines for detailed requirements. View published research articles from *PLOS ONE* for specific examples.<br><br>This statement is required for submission and **will appear in the published article** if the submission is accepted. Please make sure it is accurate.<br><br>**Funded studies**<br>Enter a statement with the following details:<br>• Initials of the authors who received each award<br>• Grant numbers awarded to each author<br>• The full name of each funder<br>• URL of each funder website<br>• Did the sponsors or funders play any role in the study design, data collection and analysis, decision to publish, or preparation of the manuscript?<br><br>Did you receive funding for this work?"	SWEDEN - SE
**Competing Interests**	The authors have declared that no competing interests exist.

Powered by Editorial Manager® and ProduXion Manager® from Aries Systems Corporation

Use the instructions below to enter a competing interest statement for this submission. On behalf of all authors, disclose any competing interests that could be perceived to bias this work—acknowledging all financial support and any other relevant financial or non-financial competing interests.<br><br>This statement is required for submission and **will appear in the published article** if the submission is accepted. Please make sure it is accurate and that any funding sources listed in your Funding Information later in the submission form are also declared in your Financial Disclosure statement.<br><br>View published research articles from *PLOS ONE* for specific examples.<br><br>**NO authors have competing interests**<br><br>Enter: *The authors have declared that no competing interests exist.*<br><br>**Authors with competing interests**<br><br>Enter competing interest details beginning with this statement:<br><br>*I have read the journal's policy and the authors of this manuscript have the following competing interests: [insert competing interests here]*<br><br>* typeset	
**Ethics Statement**<br><br>Enter an ethics statement for this submission. This statement is required if the study involved:<br><br>• Human participants<br>• Human specimens or tissue<br>• Vertebrate animals or cephalopods<br>• Vertebrate embryos or tissues	NA, this is a compilation of historical data.

Powered by Editorial Manager® and ProduXion Manager® from Aries Systems Corporation

• Field research<br><br>Write "N/A" if the submission does not require an ethics statement.<br><br>General guidance is provided below. Consult the submission guidelines for detailed instructions. **Make sure that all information entered here is included in the Methods section of the manuscript.**	

Powered by Editorial Manager® and ProduXion Manager® from Aries Systems Corporation

**Format for specific study types**<br><br>**Human Subject Research (involving human participants and/or tissue)**<br>• Give the name of the institutional review board or ethics committee that approved the study<br>• Include the approval number and/or a statement indicating approval of this research<br>• Indicate the form of consent obtained (written/oral) or the reason that consent was not obtained (e.g. the data were analyzed anonymously)<br><br>**Animal Research (involving vertebrate animals, embryos or tissues)**<br>• Provide the name of the Institutional Animal Care and Use Committee (IACUC) or other relevant ethics board that reviewed the study protocol, and indicate whether they approved this research or granted a formal waiver of ethical approval<br>• Include an approval number if one was obtained<br>• If the study involved *non-human primates*, add *additional details* about animal welfare and steps taken to ameliorate suffering<br>• If anesthesia, euthanasia, or any kind of animal sacrifice is part of the study, include briefly which substances and/or methods were applied<br><br>**Field Research**<br><br>Include the following details if this study involves the collection of plant, animal, or other materials from a natural setting:<br>• Field permit number<br>• Name of the institution or relevant body that granted permission	
**Data Availability**<br><br>Authors are required to make all data underlying the findings described fully available, without restriction, and from the time of publication. PLOS allows rare exceptions to address legal and ethical concerns. See the PLOS Data Policy and FAQ for detailed information.	Yes - all data are fully available without restriction

A Data Availability Statement describing where the data can be found is required at submission. Your answers to this question constitute the Data Availability Statement and **will be published in the article**, if accepted.<br><br>**Important:** Stating 'data available on request from the author' is not sufficient. If your data are only available upon request, select 'No' for the first question and explain your exceptional situation in the text box.<br><br>Do the authors confirm that all data underlying the findings described in their manuscript are fully available without restriction?	
**Describe where the data may be found in full sentences. If you are copying our sample text, replace any instances of XXX with the appropriate details.**<br><br>• If the data are **held or will be held in a public repository**, include URLs, accession numbers or DOIs. If this information will only be available after acceptance, indicate this by ticking the box below. For example: *All XXX files are available from the XXX database (accession number(s) XXX, XXX.)*.<br>• If the data are all contained **within the manuscript and/or Supporting Information files**, enter the following: *All relevant data are within the manuscript and its Supporting Information files.*<br>• If neither of these applies but you are able to provide **details of access elsewhere**, with or without limitations, please do so. For example:<br><br>*Data cannot be shared publicly because of [XXX]. Data are available from the XXX Institutional Data Access / Ethics Committee (contact via XXX) for researchers who meet the criteria for access to confidential data.*<br><br>*The data underlying the results presented in the study are available from (include the name of the third party*	All data and code are available on GitHub: https://github.com/DaireCarroll2023/PV_Trend_Analysis and Zenodo DOI: 10.5281/zenodo.14945453.

and contact information or URL).<br>• This text is appropriate if the data are owned by a third party and authors do not have permission to share the data.<br><br>* typeset	
Additional data availability information:	Tick here if the URLs/accession numbers/DOIs will be available only after acceptance of the manuscript for publication so that we can ensure their inclusion before publication.

Manuscript
Click here to access/download;Manuscript;Declining harbour seal abundance.docx

Declining harbour seal abundance in the Kattegat-Skagerrak and Danish Straits

Daire Carroll[1,2,*], Markus P. Ahola[3,4], Anja M. Carlsson[3], Anders Galatius[5], Kjell T. Nilssen[6], Tero Härkönen[7], and Karin C. Harding[1,2]

[1] Department of Biological and Environmental Sciences, University of Gothenburg, Gothenburg, Sweden.

[2] Gothenburg Global Biodiversity Centre, Gothenburg, Sweden.

[3] Department of Population Analysis and Monitoring, Swedish Museum of Natural History, Stockholm, Sweden.

[4] Marine Environment Research Group, Turku University of Applied Sciences, Turku, Finland.

[5] Section for Marine Mammal Research, Department of Ecoscience, Aarhus University, Roskilde, Denmark.

[6] Institute of Marine Research, Tromsø, Norway

[7] Maritimas AB, Kärna, Sweden.

*daire.carroll@bioenv.gu.se

Abstract

We present evidence that the abundance of harbour seals in the Kattegat-Skagerrak is in decline. Until recently, the Kattegat-Skagerrak harbour seal population has grown exponentially as it has recovered from historic over-hunting and two mass mortality events. This has provided an important case study for understanding the influence of environmental factors on population growth. Over recent years, deteriorating average body condition and reduced pup counts in certain colonies have indicated that the population is under stress. At the same time, there has

been an increase in hunting in the region which may compound existing environmental stressors. To determine trends in harbour seal abundance, we compile aerial survey data for the Kattegat-Skagerrak and Danish Straits between the years 2003 and 2023. With parametric modelling, we find that logistic growth models are the best fit for aerial survey data from the Kattegat and Skagerrak, while an exponential growth model is the best fit for the smaller South-Western Baltic population. Across spatial scales ranging from sub-regions, representing individual colonies, up to the combined Kattegat-Skagerrak, we determine trends by fitting non-parametric generalised additive models (GAMs) to aerial survey data. In the Kattegat-Skagerrak, we find that rate of change has declined to zero or below zero in eleven out of twelve sub-regions, with an estimated annual decline of - 408 individuals (SE = 242, $CI_{95\%}$ = [67, -882]) for the combined Kattegat-Skagerrak in 2023. This represents approximately 7 % of the mean count that year. Rate of change remained positive in the South-Western Baltic throughout the survey period at an estimated annual increase of 54 individuals (SE = 23, $CI_{95\%}$ = [99, 9]). Probable causes of declining counts in the Kattegat-Skagerrak are a combination of environmental degradation causing regionally lowered birth rates, changing haul-out behaviour, and shifts in seal distribution to less densely populated areas.

Introduction

Bioindicator species are monitored in order to track changes in environmental health [1]. European seal populations have a long history of use as bioindicators due to their responsiveness to environmental factors such as prey abundance, pollutants, and climate change [2–4]. Harbour seals (*Phoca vitulina*) in the Kattegat-Skagerrak have been the focus of extensive ecological monitoring over the past four decades [5–7]. Continued monitoring of the population's response to ecological change provides an opportunity to investigate the processes governing trends in marine mammal abundance.

The Kattegat-Skagerrak harbour seal population is largely isolated from the closest populations in the South-Western Baltic, Limfjord, and Wadden Sea [8]. Paralleling many other pinniped populations, the Kattegat-Skagerrak population declined rapidly in the early half of the twentieth century as the result of culling [5,9,10]. Sustained hunting through the middle of the century led to further decline and prevented recovery [6,11]. Harbour seals were protected from hunting in 1967 in Sweden and 1976 in Denmark [5,6]. Following this, the population grew exponentially through the 1980s and 1990s [6]. Mass mortality events, caused by outbreaks of Phocine Distemper Virus (PDV) in 1988 and 2002, killed up to 60 % of the population on each occasion. The pathogen did not become enzootic, and another mass mortality event is now possible [12,13].

The highest plausible intrinsic annual growth rate for a harbour seal population with a stable age structure is approximately 12 % [14]. During the two periods of growth following PDV outbreaks, the Kattegat-Skagerrak population displayed exponential growth of between 10.2 % and 13.6 % annually [6]. Rates of increase above 12 % are likely the result of an unstable population age structure following mass mortality events with many young females among survivors [6,14,15]. These periods of exponential growth set a baseline for the conditions of fecundity and survival which can be expected in an optimal environment without density dependence. Prior to the 2002

PDV outbreak, growth rates in the Kattegat-Skagerrak began to slow with mean counts of approximately 10,000 individuals [6]. Although the population continued to grow following the 2002 PDV outbreak, growth rates were considerably lower at 5 % and 6.5 % for the Kattegat and Skagerrak respectively [15]. These relatively low rates were likely influenced by increased mortality during 2007, which was potentially caused by exposure to algal toxins and bacterial infections, and in 2014, caused by an outbreak of influenza A(H10N7) [16–18]. Mortality during these events, however, was considerably lower than during either of the two PDV outbreaks and they do not explain continued low growth rates on their own [6,15]. This has led to speculation that the population may be experiencing resource limitation, which is further supported by observed reductions in somatic growth rates and reduced pup counts in the Skagerrak, although trends may differ elsewhere [15,19,20].

Declines in the growth of wildlife populations are often assumed to be the result of density dependence [15,21,22]. Here, intraspecific competition for resources causes a reduction in population growth. This assumption is implicit in logistic growth models [23]. Harbour seals compete for resources such as haul-out sites [24,25] and potentially for prey [26]. Intraspecific competition is not necessarily the sole cause of reduced population growth [21,27]. In parts of the world, harbour seals are known to be displaced by recovering grey seal (*Halichoerus grypus*) populations [28,29]. Grey seals remain uncommon in the Kattegat-Skagerrak [30,31]. Harbour seals are also prey to species such as white shark (*Carcharodon carcharias*) and orca (*Orcinus orca*) in parts of their range. White shark are not known to occur in the Kattegat-Skagerrak and the presence of orca is sporadic [32–34]. Reduced prey quality and abundance can limit population growth independently from predator density if nutritional needs are not being met [27,35,36]. Pollution, entanglement in fishing nets, and anthropogenic disturbance can also impose density-independent limitations on seal population growth [3,6,37,38].

Hunting by humans is a common cause of seal population decline, as evidenced by the collapse of the Kattegat-Skagerrak harbour seal population during the previous century and more recent declines in Icelandic waters [5,39]. In the Norwegian Skagerrak, between 35 and 49 harbour seals were taken annually in a quota hunt between 2015 and 2022 (data provided by the Directorate of Fisheries, Norway). In Sweden, protective hunting (allowing seals to be killed to protect fishing gear) has occurred since the early 2000s, and gradually increased to 390 individuals in 2020 [40,41]. In 2022, Sweden introduced a licensed hunt with a quota of approximately 700, although less than 50 % of this was fulfilled [41]. Licensed hunting was not been renewed in 2024 [40]. In 2018, Olsen et al. reported an average of 10 seals to be regulated through derogation shooting in Denmark [11] although this number has since increased.

The Kattegat-Skagerrak and Danish Straits have been the subject of intensive industrial fishing since the 1970s, leading to severe declines in many fish populations [42–44]. Species which once formed a large component of seal diets, such as herring (*Clupea harengus*) and Atlantic cod (*Gadus morhua*) are now less common [42,45–47]. Declines in the abundance of large predatory fish, such as Atlantic cod, have led to a large-scale ecological shift with smaller fish now dominating the ecosystem alongside abundant filamentous algae [48,49]. Harbour seals are opportunistic feeders [45,46,50] and have likely shifted their diet in response to these changes, potentially limiting population growth. In combination with these ecological shifts, runoff from agriculture and forestry has led to wide scale eutrophication and brownification [51–53]. As harbour seals exhibit high levels of site fidelity, subpopulations (often referred to as colonies) are dependent on local environmental conditions and anthropogenic management strategies which may result in variable growth rates [8,38,54,55].

Given wide scale changes in the Kattegat-Skagerrak ecosystem and indicators of declining fecundity, it is imperative for the continued use of harbour seals as a bioindicator species that changes in abundance are documented during this period of environmental perturbation. To this

end, we have compiled data from harbour seal moult surveys carried out throughout the Kattegat-Skagerrak and Danish Straits since 2003. Through a combination of parametric and non-parametric modelling, we determine trends in harbour seal counts for the combined Kattegat-Skagerrak and for subregions representing seal colonies. This work presents a valuable case study of potential growth limitation for a marine mammal population and an opportunity to begin to disentangle the influence of natural processes from environmental degradation. The results are highly relevant for the management of marine wildlife.

Methods

Data collection

The time period between 2003 and 2023 was considered, representing the years since the most recent outbreak of Phocine Distemper Virus (PDV) in 2002 [13]. Data were compiled for the Kattegat-Skagerrak and Danish Straits (Figure 1). The Kattegat-Skagerrak can be considered a metapopulation with limited exchange of individuals between subpopulations (colonies) [5,8,14]. For the purposes of management, the Kattegat and Skagerrak are often considered separately [7]. The South-Western Baltic (S.W. Baltic) was considered as a separate metapopulation based on low levels of exchange evident from genetic analysis, telemetry studies, and the fact that influenza A(H10N7) did not spread from the Kattegat into the S.W. Baltic during the 2014 outbreak but did spread into other nearby populations in the Limfjord and the Wadden Sea [8,17,54].

Aerial surveys were conducted during the peak in haul-out numbers coinciding with the annual moult in late August [56,57]. In Sweden and Denmark, haul-outs were photographed from light aircrafts [5,58]. In Norway, haul-outs were photographed using drones flown from a boat at a distance of between 800 and 1000 m. Seals were then counted from images. Counts from aerial surveys can be considered as indices of seal abundance.

For analysis, the data were divided into three sea regions (the Kattegat, the Skagerrak, and the S.W. Baltic) and 18 subregions, representing colonies (Figure 1) [5,8,15]. The structuring of harbour seal populations into subpopulations (or colonies) is well documented [8,55]. The exact definition of colony boundaries, however, is not fully resolved for the Kattegat-Skagerrak. Although harbour seals exhibit a high degree of site fidelity during the breeding season, they may display more migratory behaviour during the moulting season [38,59]. In our analysis, subregion

boundaries were based on previous work in order to facilitate comparison and explore local trends in seal abundance [5,15].

Norwegian subregions (subregion one to four, Figure 1) were surveyed between one and three times in 2016 and again in 2022. One Norwegian subregion (subregion five, Figure 1) was included in Swedish surveys. During Swedish (subregions five to 12 and parts of 17, Figure 1) and Danish (subregions 13 to 16 and parts of 17, Figure 1) surveys, subregions were surveyed between two and three times each year, except for 2004, when Danish subregions in the Kattegat were only surveyed once.

For each region (the Kattegat, the Skagerrak, and the S.W. Baltic) and each subregion (Figure 1), the sum of counts for each survey day was calculated. For each year for which more than one count was available, means of annual counts (mean count) were determined for all regions and subregions [58]. As each country conducted surveys on different days in many years, the data from each country were treated separately and annual means were summed for jointly surveyed areas. As data for only two years were available for subregions one to four (Figure 1), they were excluded from statistical analysis of trends.

Figure 1. Map of the survey area. Subregions one to eight belong to the Skagerrak region. As data for subregions one to four were only available for 2016 and 2022, they were excluded from statistical analysis of trends. Subregions nine to 16 belong to the Kattegat region. The South-Western Baltic (S.W. Baltic) (17) was treated as a separate population with just one subregion.

Analysis of trends

Parametric modelling

Exponential growth of the Kattegat-Skagerrak harbour seal population was observed following their protection from hunting in the 1970s and following the outbreak of PDV in 1988 [5,6,15]. Given indications of declining growth, two classic parametric models for population growth were fit to mean count data (*N*) using a non-linear least squares (NLS) regression. The first model represented exponential growth:

$$N_t = N_0 e^{rt} \qquad \text{Equation 1,}$$

Where N_t is mean count at time t, N_0 is initial mean count (in 2003), and r is the annual growth rate. The second model represented logistic (density dependent) growth:

$$N_t = \frac{K}{1+\left(\frac{K-N_0}{N_0}\right)e^{-\mu t}} \qquad \text{Equation 2.}$$

Where K is carrying capacity and μ is the intrinsic growth rate. Separate models were fit to data summarised at different spatial scales; ***i.*** for the combined Kattegat-Skagerrak and ***ii.*** for each region (the Kattegat, the Skagerrak, and the S.W. Baltic), taking "Year" as the explanatory variable and mean count ("Count") as response variable.

Non-parametric modelling

Parametric models (e.g., Equations 1 and 2) assume that population growth can be described by a fixed number of parameters (e.g., an intrinsic growth rate, carrying capacity, and initial population size). While they are informative when considering the underlying mechanisms controlling population growth, they also risk oversimplifying trends by, for example, assuming exponential, or density dependent growth with a fixed carrying capacity [23]. To explore trends

without assuming exponential or logistic growth and to compare trends between regions and subregions, non-parametric Generalised Additive Models (GAMs) were fit to mean count data [60,61]. Separate models were fit to population counts, summarised at three different spatial scales, taking mean count ("Count") as the response variable and "Year" as a smooth term for; **i.** The combined Kattegat-Skagerrak, **ii.** the three regions (the Kattegat, the Skagerrak, and the S.W. Baltic, including "Region" as a smooth term and an interaction between "Year" and "Region"), and **iii.** for subregions in the Kattegat-Skagerrak (including "Subregion" as a smooth term and an interaction between "Year" and "Subregion"). Rates of change were determined by calculating the first derivative of fitted GAMs.

Software and analysis

All analysis was carried out using the software R (version 4.3.2) [62] with organisation of data using the *tidyverse* package [63]. Analysis of spatial data and sorting of georeferenced observations into subregions was carried out using the *sf* package [64]. Non-linear least squares (NLS) regression was carried out using the *nls()* command with comparison of model fit based on Akaike Information Criterion (AIC). GAM fitting was carried out using the *mgcv* package using method '*REML*', an unspecified number of knots and the *tp* (thin plate regression splines) smoother within smooth terms [65] with model predictions and determination of 95 % confidence intervals ($CI_{95\%}$) made using the *investr* package [66] and estimation of first derivatives, standard errors (SEs), and 95 % confidence intervals of derivatives using the *gratia* package [67].

Code is available on GitHub: https://github.com/DaireCarroll2023/PV_Trend_Analysis and Zenodo DOI: 10.5281/zenodo.14945453.

Results

The results of aerial surveys were summarised to determine mean counts. Most subregions were surveyed each year. Danish subregions in the Kattegat were surveyed only once in 2004. This year was excluded from analysis of trends for the Kattegat and the combined Kattegat-Skagerrak. Four Norwegian colonies (colonies one to four, Figure 1) were surveyed in 2016 and 2022 only (Table S1).

Analysis of trends

For count data pooled for the combined Kattegat-Skagerrak, as well as when treating data separately for the Kattegat and Skagerrak regions, the logistic model was found to be the best fit for mean count data based on lowest AIC. For data from the S.W. Baltic, the logistic model could not be fit, leading to the conclusion that the exponential model was the best fit (Supplementary Materials Table S2 & Figure S1).

Non-parametric GAMs were fit to mean count data for the combined Kattegat-Skagerrak (AIC = 354.97, adjusted R^2 = 0.7) as well as separately for the three regions (the Kattegat, the Skagerrak and the S.W. Baltic, AIC = 987.6, adjusted R^2 = 0.96) including “Region” as a smooth term and an interaction between “Year” and “Region” (Table 2, Figure 2A).

Table 2. Outcomes of Generalised Additive Model (GAM) fitting for the combined Kattegat-Skagerrak population and including a main effect and interaction of region. An asterisk (*) indicates an interaction. Adj. R^2 = adjusted R^2. SE = standard error. EDF = effective degrees of freedom. Ref. DF = reference degrees of freedom.

**Main effects**						
**Model**	**Adj. R^2**		**Intercept**	**SE**	**t-value**	**p-value**
Kattegat-Skagerrak	0.7		12,000	340	35.25	< 0.001
Regions	0.96	Kattegat	7,979	144	55.56	< 0.001
		Skagerrak	3,827	200.4	- 20.49	< 0.001
		S.W. Baltic	855	200.4	- 35.55	< 0.001
**Approximate significance of smooth terms**						
**Model**	**Adj. R^2**		**EDF**	**Ref. DF**	***f*-value**	***p*-value**
Kattegat-Skagerrak	0.7	Year	2.43	2.78	16.63	< 0.001
Regions	0.96	Year	0.75	0.75	2.049	0.216
		Year*Kattegat	2.42	2.67	15.63	< 0.001
		Year*Skagerrak	2.23	2.56	7.81	< 0.001
		Year*S.W. Baltic	0.75	0.75	0.18	0.713

Estimated counts for the Kattegat and combined Kattegat-Skagerrak based on GAMs reached a maximum in 2017. Estimated counts for the Skagerrak reached a maximum in 2016. Estimates for the S.W. Baltic continued to increase throughout the survey period (Table S3, Figure 2A).

Figure 2. A. Mean number of harbour seals counted during moult surveys for different sea regions. Separate generalised additive models (GAMs) were fit to mean counts (points) for the combined Kattegat-Skagerrak (pink), and the three regions (the Kattegat: green, the Skagerrak: blue, and the S.W. Baltic: purple). Note the difference in y-axis scale between panels. **B.** Rates of change in population size were estimated based on the first derivative of fitted GAMs. Dashed lines represent 95 % confidence intervals of estimates. Solid black dashed lines in are placed at zero, representing no growth.

Rates of change were determined based on the first derivative of fitted GAMs (Figure 2B). At the end of the survey period, an annual decline for the Kattegat-Skagerrak population - 408 (SE = 242, $CI_{95\%}$ = [67, -882]) individuals was estimated. A decline of - 236 (SE = 111, $CI_{95\%}$ = [- 17, - 446]) was estimated in the Kattegat while a decline of - 228 (SE = 102, $CI_{95\%}$ = [- 27, - 430]) was estimated in the Skagerrak. In the S.W. Baltic population, a consistent annual increase of 54 individuals (SE = 23, $CI_{95\%}$ = [99, 9]) was estimated across the entire survey period.

GAMs were also fit to count data for individual colonies including "Subregion" as a smooth term and an interaction between "Year" and "Subregion" (AIC = 3,375.53, adjusted R^2 = 0.79, Supplementary Materials Table S4, Figure 3). Rates of change of individual subregions were also determined (Supplementary Materials Figure S2).

Figure 3. Mean number of harbour seals counted during moult surveys for subregions in the Kattegat-Skagerrak metapopulation over the last 20 years. Note an apparent change from exponential growth to linear growth and decline has occurred. A generalised additive model (GAM) was fit to mean counts (points) including subregion as a smooth term. Dashed lines represent 95 % confidence intervals of estimates. See Supplementary Materials Table S4 for details on model fitting.

236

Discussion

We have summarised the two most recent decades of harbour seal aerial survey data for the Kattegat-Skagerrak area and South-Western Baltic (S.W. Baltic). Counts in both the Kattegat and the Skagerrak reached a maximum around 2017 and have since declined. This contrasts with historical exponential growth observed between the 1980s and early 2000s [6]. The estimated count in 2023 for the combined Kattegat-Skagerrak (12,507, $CI_{95\%}$ = [13,456, 11,558]) was higher than the mean counts of approximately 10,000 individuals prior to the 2002 PDV outbreak [6]. Heide-Jorgensen and Härkönen (1988) estimated the size of the Kattegat-Skagerrak harbour seal population in 1890 to number 16,500 based on bounty statistics [5,6]. At the time, the population was already under considerable hunting pressure [5]. Although they should be considered as coarse estimates only, previous work has assumed the hauled out fraction of the Kattegat-Skagerrak harbour seal population observed during moult surveys to be between 57 % and 65 % [6,15]. Assuming either value would indicate that the Kattegat-Skagerrak harbour seal population in 2023 was close to 20,000, considerably larger than the 1890 estimate.

Contrastingly, population counts continue to increase for the nearby S.W. Baltic population. Relative to the Kattegat-Skagerrak, counts in the S.W. Baltic in the 1970s were low (around 100). Hunting pressure was also high relative to the population size in the years leading up to protection from hunting [6,68]. As a result of their relatively slow intrinsic population growth rate, harbour seal populations today are still recovering from over-hunting in the previous century [69,70]. A probable explanation for the continued growth of the S.W. Baltic population is that it was close to extinction when protected from hunting and that population density hence remains low relative to the Kattegat-Skagerrak.

Harbour seals show high levels of breeding site fidelity and it is unlikely that significant levels of emigration out of the Kattegat-Skagerrak metapopulation have occurred over the past twenty

years [8,54]. This is especially true considering the lack of growth in the nearby Wadden Sea population and the relatively small sizes of the (genetically distinct) closest populations in the Baltic Sea and Limfjord [6,69]. Limited migration does likely occur between individual colonies in the Kattegat-Skagerrak [55].

Over recent years, harbour seals have recolonised the South Funen Archipelago and Little Belt area of the Danish Straits, with average moult counts of 155 seals between 2021 and 2023 [70]. Although they could not be included in modelling, four Norwegian subregions (Farsund, Tvedestrand, Kragerø, and Farsund) showed an increase in total mean count from 365 to approximately 658 between 2016 and 2022. The long interval between surveys in Norway prevented subsequent analysis of trends. It is possible that increases along the Norwegian East Coast are driven by movement from the more densely populated Swedish West Coast. Although counts in both the Norwegian colonies and the South Funen Archipelago and Little Belt are small relative to the total Kattegat-Skagerrak population, they may become increasingly important in the future.

Based on non-parametric modelling, eleven out of twelve subregions in the Kattegat-Skagerrak displayed either a negative trend or little to no increase in mean count. The one subregion to display a positive trend, Marstrand, was bordered by the two subregions with the largest absolute rates of decline (Lysekil and Onsala) and may be influenced by local immigration. The exact delineation of colony boundaries within the Kattegat-Skagerrak is currently unknown. The subregions employed in the present study were based on assumed colonies used in previous work [5,15]. Many of these were defined based on geographic features, such as isolated islands or archipelagos (e.g., Anholt and Koster). For Sweden in particular, subregions also included large sections of coastline, which are unlikely to represent true colonies. Nonetheless, our analysis indicates that trends are largely consistent throughout the Kattegat-Skagerrak, with no single

subregion driving apparent changes in abundance. Future work could aim to more clearly define colony boundaries to improve the assessment of localised trends in seal abundance [8,55]

The demographic structure of the hauled-out fraction of a harbour seal population changes throughout the moult due to differential behaviour of age- and sex-classes [71]. Haul-out frequency is also influenced by environmental factors such as temperature and wind speed [57,72,73]. It is possible that increased harbour seal population density or changes in demographic structure have altered haul-out behaviour in the Kattegat-Skagerrak, potentially lowering the fraction of seals observed during surveys [24,25,74]. Additionally, disturbance of haul-out sites by vessels, and of seal foraging activities by offshore construction may influence trends [37,75]. In 2020, there was a reported increase in recreational use of the coast by the Swedish public [76]. This may partially account for particularly low counts that year. In future, targeted surveys using telemetry tags and/or individual identification methods should be used to study changes in haul-out behaviour relative to previous work [54,71]. Recent advances in drone-based survey methods could also be used to determine patterns in haul-out behaviour between age classes [20,77,78].

While they likely play a role, it is unlikely that declining counts are an artefact of behavioural changes alone when considered in combination with independent data on localised reductions in pup counts and somatic growth in the Skagerrak [19,20]. The shift from exponential growth to an equilibrium is predicted in population ecology theory as intraspecific competition is increased and one or more resources becomes limiting [36,79]. Based on logistic modelling, we have estimated carrying capacities for the Kattegat-Skagerrak region as well as its subregions. These theoretical maximum values can be used in predictive modelling to assess the potential impact of management decisions on population viability [8,15,22]. Logistic models, however, assume a static ecosystem with a fixed carrying capacity [23]. A number of harbour seal populations around the world have experienced well documented shifts from growth to decline [39,80,81]. Exploring

trends through more flexible non-parametric modelling is therefore of value, as a decline may indicate an environmental shift rather than the population reaching an equilibrium based on intra-specific competition.

Average annual adult survival in pinnipeds is generally high (often > 95 % in harbour seals) with variation in population growth being primarily driven by birth rates and juvenile survival [14,82–84]. Given documented shifts in the Kattegat-Skagerrak ecosystem and the abundance of harbour seal prey species [42–44], it seems likely that food limitation, leading to lower birth rates, and potentially lower juvenile survival, is a contributing factor to declining counts. Such shifts in diet are likely driven by anthropogenic activities, such as overfishing, forestry, and construction, which have led to widescale environmental degradation [42,43,51–53]. The effects of dietary changes could be investigated through comparative studies of the composition and nutritional value of diet and seal body condition between the Kattegat-Skagerrak and the growing South-Western Baltic population. Given the long history of ecological monitoring in the Kattegat-Skagerrak, there is also the opportunity to compare modern [50] and historical [45,46] diet composition.

A key question is the degree to which intraspecific competition is driving food limitation (density dependent limitation). Alternatively, individual seals may be struggling to meet their nutrient requirements irrespective of competition (density independent limitation). During density dependent limitation, reductions in population size are expected to result in an increased growth rate [35,36,85]. In this case, mass mortality events, such as the 1988 and 2002 PDV outbreaks, will result in a return to exponential growth and eventual recovery of the population [13]. During density independent limitation, lower growth rates will be maintained even if a large portion of the population is killed. This is true both for natural mortality events and for anthropogenic mortality, such as hunting, putting the population at greater risk of overexploitation. A mixture of density dependent and independent control of population growth is likely.

During the late 2010s, there was an increase in protective hunting of harbour seals in Sweden [15,40,41]. Increases in anthropogenic hunting are one possible cause of reduced population growth in the Kattegat-Skagerrak, especially when combined with additional stressors [15]. It is unlikely, however, that hunting alone is responsible for reduced population growth given the negative trends observed in Danish colonies, where hunting pressure is negligible. Levels of bycatch in the Kattegat-Skagerrak are poorly quantified and represent an additional source of anthropogenic mortality for harbour seals [86,87]. At present, there is no reason to believe that bycatch levels have increased in recent years, and they may even be in decline as the use of passive fishing gear in the Kattegat Skagerrak has declined [88,89].

Conclusion

We have presented evidence of declining abundance of harbour seals in the Kattegat-Skagerrak. This contrasts with the nearby population in the South-Western Baltic Sea which continues to increase (by approximately 50 animals per year), likely due to its lower population density. The changes in seal abundance we have reported present an opportunity to study the mechanisms by which environmental factors regulate population growth and animal behaviour. While changes in haul-out behaviour may contribute to the apparent decline in counted harbour seals, it seems likely that movement of seals within the metapopulation and nutrient limitation are also driving real shifts in seal abundance. Future work should investigate the role of factors such as migration, intraspecific competition, decreases in the abundance or quality of prey, availability of pupping sites, and hunting in determining population growth rates. Annual monitoring of harbour seal abundance represents a large investment of time and resources by national authorities. This effort is justified as the state of marine mammal populations are indicators of overall environmental health. The warning signs presented by a decrease in seal abundance should not be ignored by researchers or ecosystem managers. The management of harbour seals should be updated to

account for a lower growth potential, for example by easing hunting pressure or improving seal sanctuary design.

Data availability statement

All data and code are available on GitHub: https://github.com/DaireCarroll2023/PV_Trend_Analysis and Zenodo DOI: 10.5281/zenodo.14945453.

Acknowledgements

Swedish surveys were funded by the Swedish Environmental Protection Agency, and the Swedish Agency for Marine and Water Management. Danish surveys were funded by the Danish Environmental Protection Agency. The authors thank the pilots and observers who worked on the surveys. DC thanks the Wild Animal Initiative Fellowship, grant numbers F-2023-00005 and the Swedish Environmental Protection Agency through the Environmental research fund and the Swedish Research Council Formas, grant number (2024-00147). KCH was supported by the European Union (HORIZON-CL6-2023-BIODIV-01-101135307). The authors thank J. Harvey-Carroll for her feedback on the initial manuscript.

Author contributions

Conceptualization: DC, TH, and KCH. Data Curation: DC, MPA, AMC, AG, JAH, KTN. Data Collection: MPA, AC, AG, KTN. Formal Analysis: DC. Writing – Original Draft Preparation: DC. Writing – Review & Editing: All authors.

References

1. Burger J. Bioindicators: Types, Development, and Use in Ecological Assessment and Research. Environmental Bioindicators. 2006 Jan;1(1):22–39.

2. Bäcklin BM, Moraeus C, Roos A, Eklöf E, Lind Y. Health and age and sex distributions of Baltic grey seals (*Halichoerus grypus*) collected from bycatch and hunt in the Gulf of Bothnia. ICES Journal of Marine Science. 2011 Jan 1;68(1):183–8.

3. Bergman A. Health condition of the Baltic grey seal (*Halichoerus grypus*) during two decades. APMIS. 1999 Mar;107(1–6):270–82.

4. Sundqvist L, Harkonen T, Svensson CJ, Harding KC. Linking Climate Trends to Population Dynamics in the Baltic Ringed Seal: Impacts of Historical and Future Winter Temperatures. AMBIO. 2012 Dec;41(8):865–72.

5. Heide-Jørgensen MP, Härkönen TJ. Rebuilding seal stocks in the Kattegat-Skagerrak. Marine Mammal Sci. 1988 Jul;4(3):231–46.

6. Olsen MT, Andersen SM, Teilmann J, Dietz R, Edrén SMC, Linnet A, et al. Status of the harbour seal (*Phoca vitulina*) in Southern Scandinavia. NAMMCOSP. 2010;8:77.

7. ICES. Working Group on Marine Mammal Ecology (WGMME). ICES Scientific Reports; 2024 [cited 2025 Feb 10]. Available from: https://ices-library.figshare.com/articles/report/Working_Group_on_Marine_Mammal_Ecology_WGMME_/26997367/1

8. Olsen MT, Andersen LW, Dietz R, Teilmann J, Härkönen T, Siegismund HR. Integrating genetic data and population viability analyses for the identification of harbour seal (*Phoca vitulina*) populations and management units. Molecular Ecology. 2014 Feb;23(4):815–31.

9. Bowen WD, Lidgard D. Marine mammal culling programs: review of effects on predator and prey populations. Mammal Review. 2013;43(3):207–20.

10. Magera AM, Mills Flemming JE, Kaschner K, Christensen LB, Lotze HK. Recovery Trends in Marine Mammal Populations. Stergiou KI, editor. PLoS ONE. 2013 Oct 30;8(10):e77908.

11. Olsen M, Galatius A, Härkönen T. The history and effects of seal-fishery conflicts in Denmark. Mar Ecol Prog Ser. 2018 May 14;595:233–43.

12. Dietz R, Härkönen T, Heide-Jørgensen MP. Mass deaths of harbor seals (*Phoca vitulina*) in Europe. Ambio. 1989;18:258–64.

13. Härkönen T, Dietz R, Reijnders P, Teilmann J, Harding K, Hall A, et al. The 1988 and 2002 phocine distemper virus epidemics in European harbour seals. Dis Aquat Org. 2006;68:115–30.

14. Härkönen T, Harding KC, Heide-Jørgensen MP. Rates of increase in age-structured populations: a lesson from the European harbour seals. Can J Zool. 2002 Sep 1;80(9):1498–510.

15. Silva WTAF, Bottagisio E, Härkönen T, Galatius A, Olsen MT, Harding KC. Risk for overexploiting a seemingly stable seal population: influence of multiple stressors and hunting. Ecosphere. 2021 Jan;12(1).

16. Härkönen T, Bäcklin BM, Barrett T, Bergman A, Corteyn M, Dietz R, et al. Mass mortality in harbour seals and harbour porpoises caused by an unknown pathogen. Veterinary Record. 2008 Apr;162(17):555–6.

17. Bodewes R, Zohari S, Krog JS, Hall MD, Harder TC, Bestebroer TM, et al. Spatiotemporal Analysis of the Genetic Diversity of Seal Influenza A(H10N7) Virus, Northwestern Europe. Schultz-Cherry S, editor. J Virol. 2016 May;90(9):4269–77.

18. Mollerup IM, Bjørneset J, Krock B, Jensen TH, Galatius A, Dietz R, et al. Did algal toxin and Klebsiella infections cause the unexplained 2007 mass mortality event in Danish and Swedish marine mammals? Science of The Total Environment. 2024 Mar;914:169817.

19. Harding KC, Salmon M, Teilmann J, Dietz R, Harkonen T. Population Wide Decline in Somatic Growth in Harbor Seals—Early Signs of Density Dependence. Front Ecol Evol. 2018 May 11;6:59.

20. Infantes E, Carroll D, Silva WTAF, Härkönen T, Edwards SV, Harding KC. An automated work-flow for pinniped surveys: A new tool for monitoring population dynamics. Frontiers in Ecology and Evolution. 2022;10.

21. Rotella JJ. Patterns, sources, and consequences of variation in age-specific vital rates: Insights from a long-term study of Weddell seals. Journal of Animal Ecology. 2022 Dec 18;1365-2656.13870.

22. Carroll D, Ahola MP, Carlsson AM, Sköld M, Harding KC. 120-years of ecological monitoring data shows that the risk of overhunting is increased by environmental degradation for an isolated marine mammal population: The Baltic grey seal. Journal of Animal Ecology. 2024 Mar 26;1365-2656.14065.

23. Tsoularis A, Wallace J. Analysis of logistic growth models. Mathematical Biosciences. 2002 Jul;179(1):21–55.

24. Neumann DR. Agonistic behavior in harbor seals (*Phoca vitulina*) in relation to the availability of haul-out space. Marine Mammal Science. 1999 Apr;15(2):507–25.

25. Honeywell AF, Maher CR. Intensity, rate, and outcome of agonistic interactions in harbor seals (*Phoca vitulina concolor*) vary with density on haul-out ledges. JMAMMA. 2016 Sep 26;gyw155.

26. Wilson RP, Liebsch N, Gómez-Laich A, Kay WP, Bone A, Hobson VJ, et al. Options for modulating intra-specific competition in colonial pinnipeds: the case of harbour seals (*Phoca vitulina*) in the Wadden Sea. PeerJ. 2015 Jun 9;3:e957.

27. Silva WTAF, Harding KC, Marques GM, Bäcklin BM, Sonne C, Dietz R, et al. Life cycle bioenergetics of the gray seal (*Halichoerus grypus*) in the Baltic Sea: Population response to environmental stress. Environment International. 2020 Dec;145:106145.

28. Bowen WD, McMillan J, Mohn R. Sustained exponential population growth of grey seals at Sable Island, Nova Scotia. ICES Journal of Marine Science. 2003 Jan 1;60(6):1265–74.

29. Svensson CJ. Seal dynamics on the Swedish west coast: Scenarios of competition as Baltic grey seal intrude on harbour seal territory. Journal of Sea Research. 2012 Jul;71:9–13.

30. Galatius A, Teilmann J, Dähne M, Ahola M, Westphal L, Kyhn LA, et al. Grey seal *Halichoerus grypus* recolonisation of the southern Baltic Sea, Danish Straits and Kattegat. Wildlife Biology. 2020 Dec;2020(4):1–10.

31. Galatius A, Olsen MT, Allentoft-Larsen M, Balle JD, Kyhn LA, Sveegaard S, et al. Evidence of distribution overlap between Atlantic and Baltic grey seals. J Mar Biol Ass. 2024;104:e30.

32. Lucas Z, Stobo WT. Shark-inflicted mortality on a population of harbour seals (*Phoca vitulina*) at Sable Island, Nova Scotia. Journal of Zoology. 2000 Nov;252(3):405–14.

33. Ashley EA, Olson JK, Adler TE, Raverty S, Anderson EM, Jeffries S, et al. Causes of Mortality in a Harbor Seal (Phoca vitulina) Population at Equilibrium. Front Mar Sci. 2020 May 13;7:319.

34. Hague EL, McCaffrey N, Shucksmith R, McWhinnie L. Predation in the Anthropocene: Harbour Seal (Phoca vitulina) Utilising Aquaculture Infrastructure as Refuge to Evade Foraging Killer Whales (Orcinus orca). Aquat Mamm. 2022 Jul 15;48(4):380–93.

35. Trites AW, Donnelly CP. The decline of Steller sea lions *Eumetopias jubatus* in Alaska: a review of the nutritional stress hypothesis: Nutritional stress hypothesis. Mammal Review. 2003 Mar;33(1):3–28.

36. Rotella JJ, Link WA, Nichols JD, Hadley GL, Garrott RA, Proffitt KM. An evaluation of density-dependent and density-independent influences on population growth rates in Weddell seals. Ecology. 2009 Apr;90(4):975–84.

37. Thompson PM, Hastie GD, Nedwell J, Barham R, Brookes KL, Cordes LS, et al. Framework for assessing impacts of pile-driving noise from offshore wind farm construction on a harbour seal population. Environmental Impact Assessment Review. 2013 Nov;43:73–85.

38. Blanchet MA, Vincent C, Womble JN, Steingass SM, Desportes G. Harbour Seals: Population Structure, Status, and Threats in a Rapidly Changing Environment. Oceans. 2021 Jan 5;2(1):41–63.

39. Granquist SM. The Icelandic harbour seal (Phoca vitulina) population: trends over 40 years (1980–2020) and current threats to the population. NAMMCOSP. 2022 Dec 20;12.

40. Naturvårdsverket. Jakt på knubbsäl [Internet]. 2024 [cited 2024 Jun 25]. Available from: https://www.naturvardsverket.se/lagar-och-regler/beslut/sal/knubbsal/

41. Härkönen T, Lunneryd SG, Harding KC. Kunskapssammanställning om reproduktionsperioder och andra störningskänsliga perioder hos gråsäl, knubbsäl och vikaresäl. Naturvårdsverket; 2023.

42. Bartolino V, Cardinale M, Svedäng H, Linderholm HW, Casini M, Grimwall A. Historical spatiotemporal dynamics of eastern North Sea cod. Marshall CT, editor. Can J Fish Aquat Sci. 2012 May;69(5):833–41.

43. Capuzzo E, Lynam CP, Barry J, Stephens D, Forster RM, Greenwood N, et al. A decline in primary production in the North Sea over 25 years, associated with reductions in zooplankton abundance and fish stock recruitment. Global Change Biology. 2018 Jan;24(1).

44. ICES. Report of the Working Group on the assessment of demersal stocks in the North Sea and Skagerrak (WGNSSK). 2018;4(43).

45. Härkönen T. Seasonal and regional variations in the feeding habits of the harbour seal, *Phoca vitulina*, in the Skagerrak and the Kattegat. Journal of Zoology. 1987 Nov;213(3):535–43.

46. Olsen M, Bjørge A. Seasonal and regional variations in the diet of harbour seal in Norwegian waters. In: Developments in Marine Biology. Elsevier; 1995. p. 271–85. Available from: https://linkinghub.elsevier.com/retrieve/pii/S0163699506800302

47. Dickey-Collas M, Nash RDM, Brunel T, Van Damme CJG, Marshall CT, Payne MR, et al. Lessons learned from stock collapse and recovery of North Sea herring: a review. ICES Journal of Marine Science. 2010 Dec 1;67(9):1875–86.

48. Pihl L, Isaksson I, Wennhage H, Moksnes PO. Recent increase of filamentous algae in shallow Swedish bays: Effects on the community structure of epibenthic fauna and fish. Netherlands Journal of Aquatic Ecology. 1995 Dec;29(3–4):349–58.

49. Svedäng H. The inshore demersal fish community on the Swedish Skagerrak coast: regulation by recruitment from offshore sources. ICES Journal of Marine Science. 2003 Feb;60(1):23–31.

50. Sørlie M, Nilssen KT, Bjørge A, Freitas C. Diet composition and biomass consumption of harbour seals in Telemark and Aust-Agder, Norwegian Skagerrak. Marine Biology Research. 2020 Apr 20;16(4):299–310.

51. Boström C, Baden S, Bockelmann A, Dromph K, Fredriksen S, Gustafsson C, et al. Distribution, structure and function of Nordic eelgrass (*Zostera marina*) ecosystems: implications for coastal management and conservation. Aquatic Conservation. 2014 Jun;24(3):410–34.

52. Skogen MD, Eilola K, Hansen JLS, Meier HEM, Molchanov MS, Ryabchenko VA. Eutrophication status of the North Sea, Skagerrak, Kattegat and the Baltic Sea in present and future climates: A model study. Journal of Marine Systems. 2014 Apr;132:174–84.

53. Opdal AF, Andersen T, Hessen DO, Lindemann C, Aksnes DL. Tracking freshwater browning and coastal water darkening from boreal forests to the Arctic Ocean. Limnol Oceanogr Letters. 2023 Aug;8(4):611–9.

54. Dietz R, Teilmann J, Andersen SM, Rigét F, Olsen MT. Movements and site fidelity of harbour seals (*Phoca vitulina*) in Kattegat, Denmark, with implications for the epidemiology of the phocine distemper virus. ICES Journal of Marine Science. 2013 Jan 1;70(1):186–95.

55. Liu X, Rønhøj Schjøtt S, Granquist SM, Rosing-Asvid A, Dietz R, Teilmann J, et al. Origin and expansion of the world's most widespread pinniped: Range-wide population genomics of the harbour seal (*Phoca vitulina*). Molecular Ecology. 2022 Mar;31(6):1682–99.

56. Härkönen T, Hårding KC, Lunneryd SG. Age- and sex-specific behaviour in harbour seals *Phoca vitulina* leads to biased estimates of vital population parameters. Journal of Applied Ecology. 1999 Oct;36(5):825–41.

57. Galatius A, Engbo SG, Teilmann J, Beest FMV. Using environmental variation to optimize aerial surveys of harbour seals. Camphuysen K, editor. ICES Journal of Marine Science. 2021 Aug 20;78(4):1500–7.

58. Teilmann J, Rigét F, Harkonen T. Optimizing survey design for Scandinavian harbour seals: population trend as an ecological quality element. ICES Journal of Marine Science. 2010 Jul 1;67(5):952–8.

59. Womble JN, Gende SM. Post-Breeding Season Migrations of a Top Predator, the Harbor Seal (*Phoca vitulina richardii*), from a Marine Protected Area in Alaska. Fahlman A, editor. PLoS ONE. 2013 Feb 14;8(2):e55386.

60. Fewster RM, Buckland ST, Siriwardena GM, Baillie SR, Wilson JD. Analysis of population trends for farmland birds using generalized additive models. Ecology. 2000 Jul;81(7):1970–84.

61. Hastie TJ, editor. Statistical Models in S. 1st ed. Routledge; 2017.

62. R Core Team. R: A language and environment for statistical computing. [Internet]. Vienna, Austria: R Foundation for Statistical Computing; 2017. Available from: https://www.R-project.org/.

63. Wickham H, Averick M, Bryan J, Chang W, McGowan L, François R, et al. Welcome to the Tidyverse. JOSS. 2019 Nov 21;4(43):1686.

64. Pebesma E. Simple Features for R: Standardized Support for Spatial Vector Data. The R Journal. 2018;10(1):439.

65. Wood SN. Generalized Additive Models: An Introduction with R [Internet]. 2nd ed. Chapman and Hall/CRC; 2017 [cited 2024 May 2]. Available from: https://www.taylorfrancis.com/books/9781498728348

66. Greenwell B M, Kabban C M ,Schubert. investr: An R Package for Inverse Estimation. The R Journal. 2014;6(1):90.

67. Simpson G. gratia: Graceful ggplot-Based Graphics and Other Functions for GAMs Fitted using mgcv [Internet]. 2024. Available from: https://gavinsimpson.github.io/gratia/

68. Joensen AH, Søndergaard NO, Hansen EB. Occurrence of seals and seal hunting in Denmark. Danish Review of Game Biology. 1976;10(1):1–20.

69. Brasseur SMJM, Reijnders PJH, Cremer J, Meesters E, Kirkwood R, Jensen LF, et al. Echoes from the past: Regional variations in recovery within a harbour seal population. Serrano Ferron E, editor. PLoS ONE. 2018 Jan 3;13(1):e0189674.

70. Galatius A, Dietz R, Olsen MT, Teilmann J. Recolonisation of former habitat by harbour seals in southern Denmark despite intense anthropogenic activity. J Mar Biol Ass. 2024;104:e85.

71. Härkönen T, Harding KC. Spatial structure of harbour seal populations and the implications thereof. Can J Zool. 2001 Dec 1;79(12):2115–27.

72. Simpkins MA, Withrow DE, Cesarone JC, Boveng PL. Stability in the proportion of harbor seals hauled out under locally ideal conditions. Marine Mammal Science. 2003 Oct;19(4):791–805.

73. Van Meurs E, Moland E, Bjørge A, Freitas C. Haulout Patterns of Harbour Seal Colonies in the Norwegian Skagerrak, as Monitored through Time-Lapse Camera Surveys. Diversity. 2024 Jan 5;16(1):38.

74. Cunningham L. Using Computer-Assisted Photo-Identification and Capture-Recapture Techniques to Monitor the Conservation Status of Harbour Seals (*Phoca vitulina*). aquatic mammals. 2009 Sep 1;35(3):319–29.

75. Jones EL, Hastie GD, Smout S, Onoufriou J, Merchant ND, Brookes KL, et al. Seals and shipping: quantifying population risk and individual exposure to vessel noise. González-Suárez M, editor. Journal of Applied Ecology. 2017 Dec;54(6):1930–40.

76. Hansen AS, Beery T, Fredman P, Wolf-Watz D. Outdoor recreation in Sweden during and after the COVID-19 pandemic – management and policy implications. Journal of Environmental Planning and Management. 2023 Jun 7;66(7):1472–93.

77. Carroll D, Infantes E, Pagan EV, Harding KC. Approaching a population-level assessment of body size in pinnipeds using drones, an early warning of environmental degradation. Remote Sens Ecol Conserv. 2024 Jun 25;rse2.413.

78. Hoekendijk JPA, Grundlehner A, Brasseur S, Kellenberger B, Tuia D, Aarts G. Stay close, but not too close: aerial image analysis reveals patterns of social distancing in seal colonies. R Soc open sci. 2023 Aug;10(8):230269.

79. Caswell H. Matrix population models. Vol. 1. Sinauer Sunderland, MA; 2000.

80. Bowen W, Ellis SL, Iverson SJ, Boness DJ. Maternal and newborn life-history traits during periods of contrasting population trends: implications for explaining the decline of harbour seals (*Phoca vitulina*), on Sable Island. Journal of Zoology. 2003 Oct;261(2):155–63.

81. Lonergan M, Duck CD, Thompson D, Mackey BL, Cunningham L, Boyd IL. Using sparse survey data to investigate the declining abundance of British harbour seals. Journal of Zoology. 2007 Mar;271(3):261–9.

82. Hall AJ, McConnell BJ, Barker RJ. Factors affecting first-year survival in grey seals and their implications for life history strategy: *First-year survival in grey seals*. Journal of Animal Ecology. 2001 Jan;70(1):138–49.

83. Thomas L, Russell DJF, Duck CD, Morris CD, Lonergan M, Empacher F, et al. Modelling the population size and dynamics of the British grey seal. Aquatic Conserv: Mar Freshw Ecosyst. 2019 Sep;29(S1):6–23.

84. Kjellqwist S. Trends in age-composition, growth and reproductive parameters of Barents Sea harp seals, Phoca groenlandica. ICES Journal of Marine Science. 1995 Apr;52(2):197–208.

85. Sæther BE. Environmental stochasticity and population dynamics of large herbivores: a search for mechanisms. Trends in Ecology & Evolution. 1997 Apr;12(4):143–9.

86. HELCOM. Number of drowned mammals and waterbirds in fishing gear. HELCOM core indicator report. [Internet]. 2023 [cited 2024 Nov 5]. Report No.: ISSN 2343-2543. Available from: https://indicators.helcom.fi/indicator/bycatch/

87. Bjørge A, Moan A, Nilssen KT. Bycatch of harbour and grey seals in Norway. 2017. (Working document to NAMMCO Working Group on Bycatch). Report No.: SC/24/BYCWG/07.

88. Swedish Agency for Marine and Water Managment. Swedish Fleet Capacity Report 2020 [Internet]. 2021. Available from: https://oceans-and-fisheries.ec.europa.eu/system/files/2021-09/2020-fleet-capacity-report-sweden_en.pdf

89. Statistics Denmark. Danish vessels by region, unit, type of vessels, length and tonnage - StatBank Denmark - data and statistics [Internet]. 2025 [cited 2025 Feb 24]. Available from: https://www.statistikbanken.dk/statbank5a/SelectVarVal/Define.asp?Maintable=FISK1&PLanguage=1

Figure 1

Click here to access/download;Figure;Figure 1.tif

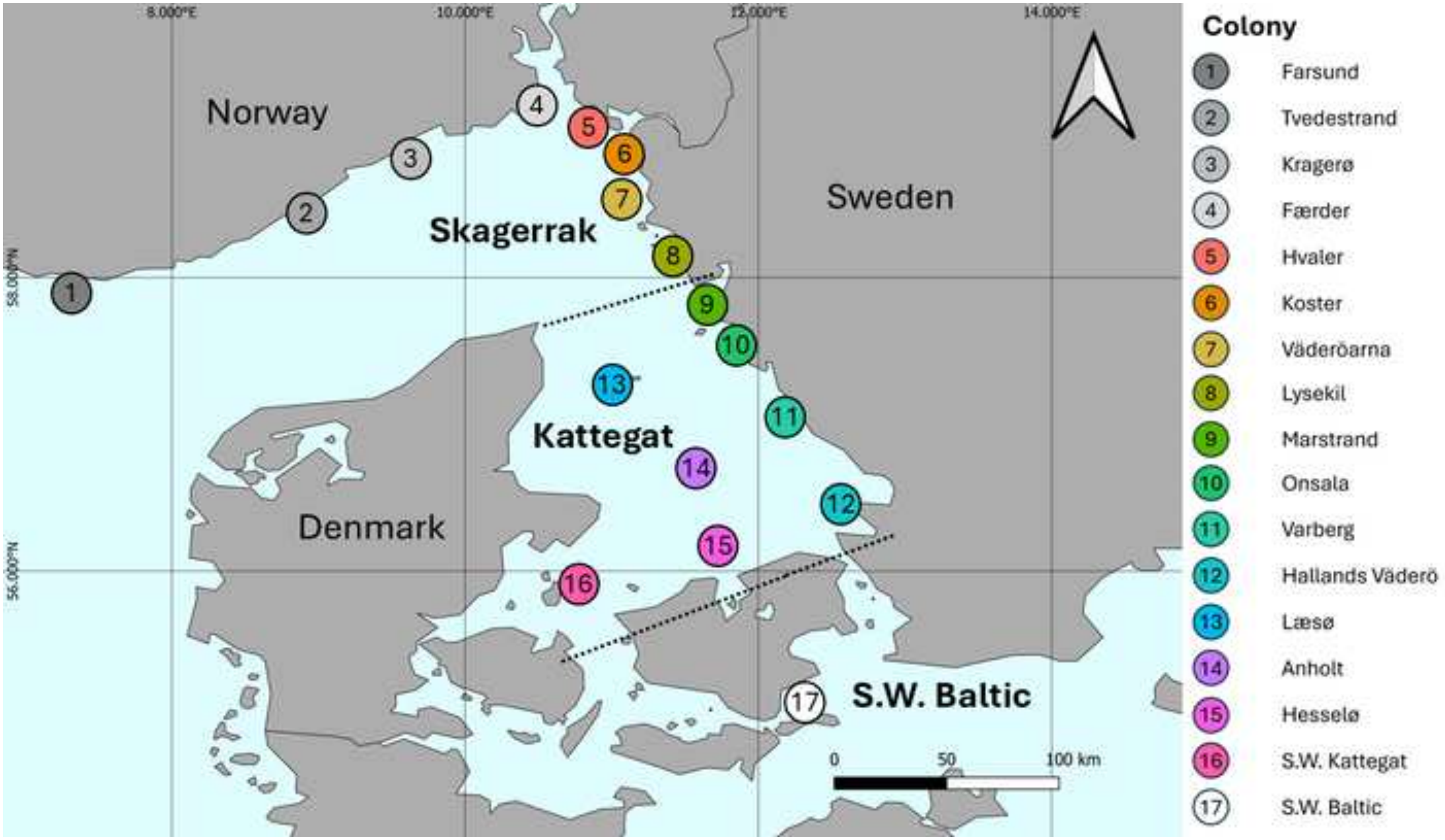

Figure 2

Click here to access/download;Figure;Figure 2.tif

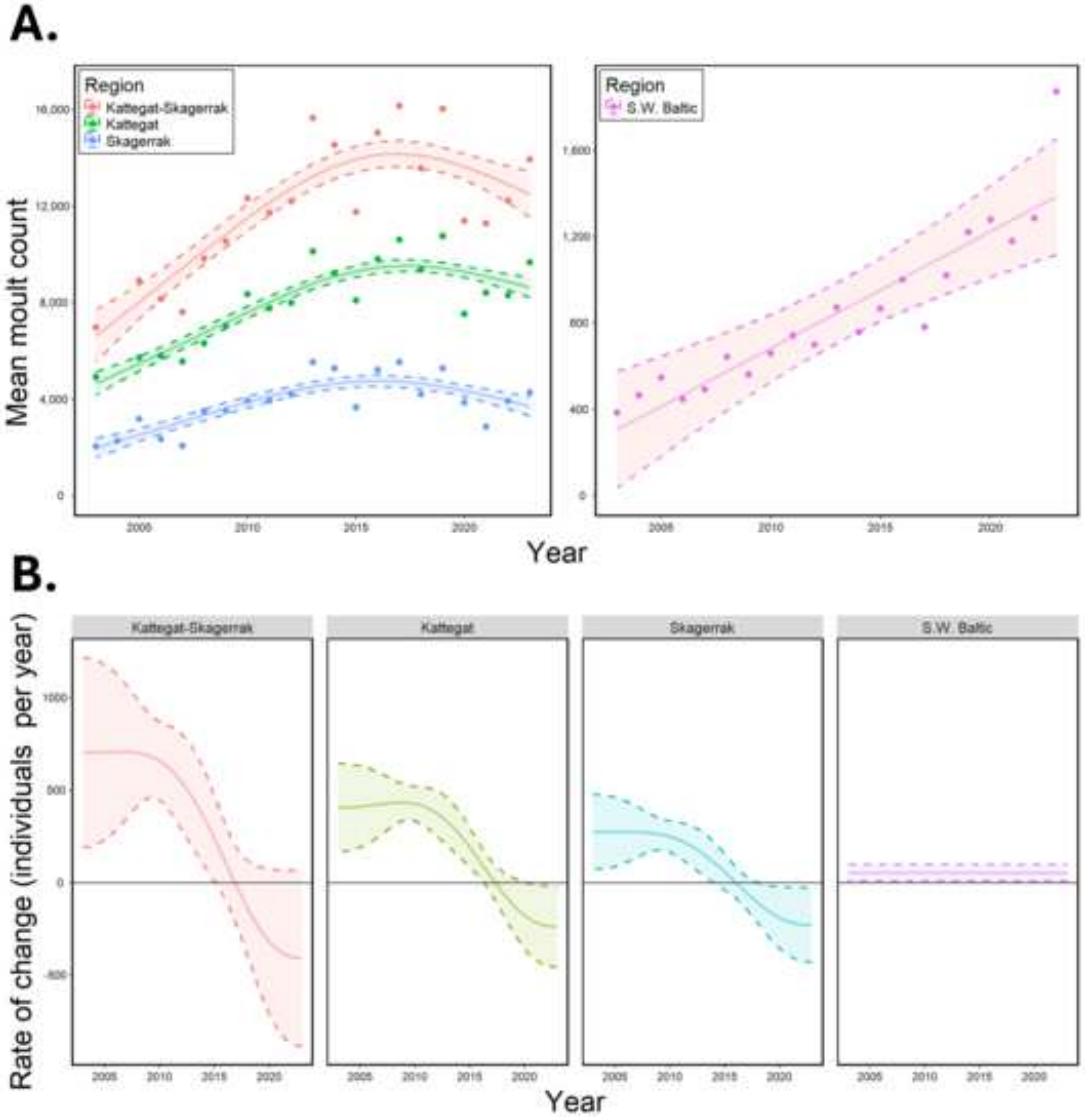

Figure 3

Click here to access/download;Figure;Figure 3.tif

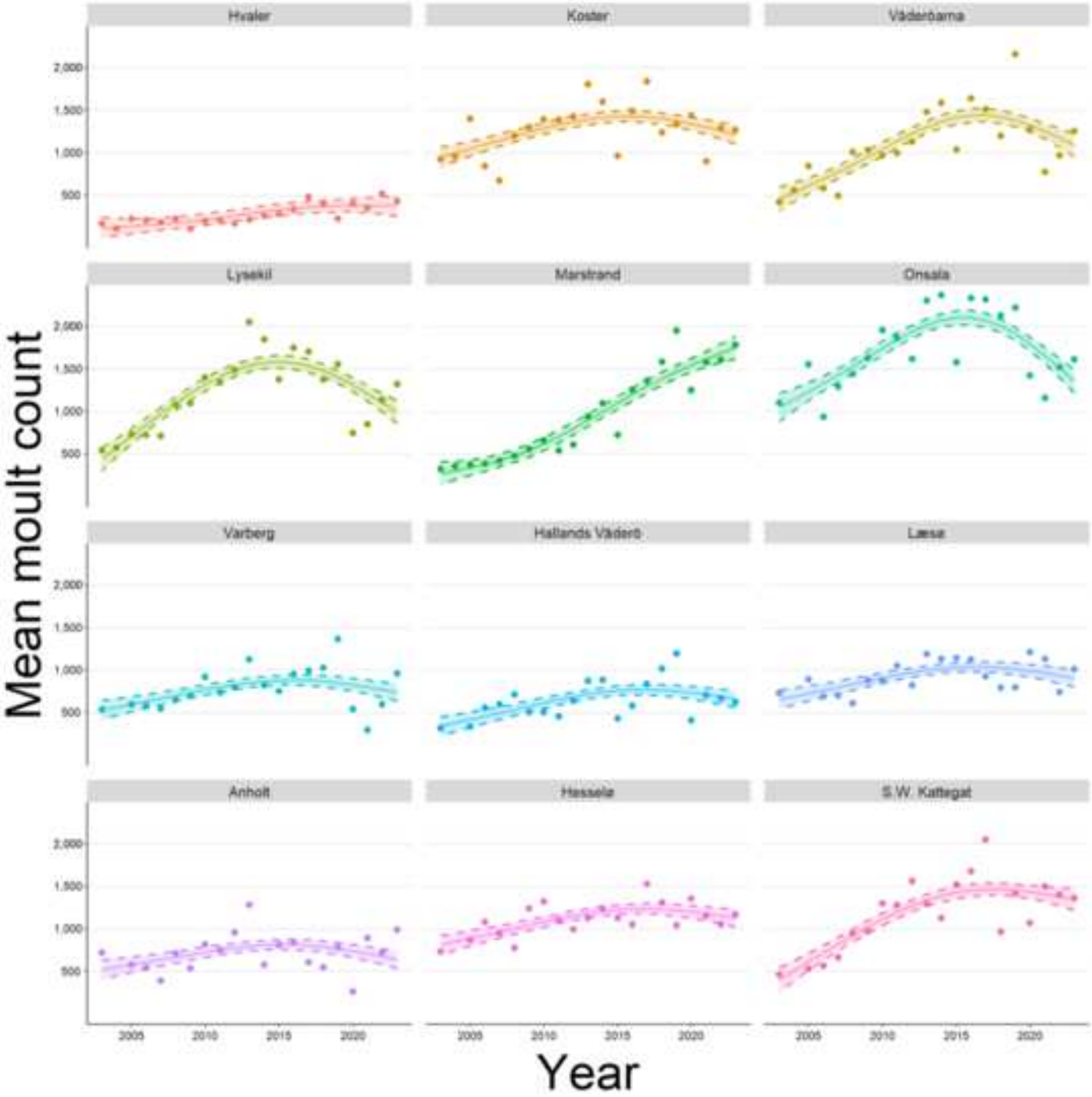

Supplamentary materials

Click here to access/download
Supporting Information
Supplementary Materials.docx

GitHub repository to be made public on acceptance of manuscript.

Click here to access/download
Supporting Information - Compressed/ZIP File Archive
GitHub.zip
