# Supplementary material for: Declining harbour seal abundance in a previously recovering meta-population
Source: PLoS One. 2025 Jun 30;20(6):e0326933. doi: 10.1371/journal.pone.0326933 (PMC12208499; doi:10.1371/journal.pone.0326933)
Supplement: Attachment — Submitted filename: Revision Responses.docx [file pone.0326933.s011.docx]

## Responses to Editor

Many thanks for your comments as well as organising a prompt and helpful review of our manuscript. We have now made changes in response to both reviewer’s comments which we believe brings it in line with PLOS ONE’s publication criteria. Below, we detail the changes that have been made.

We have updated formatting in our main text and supplementary materials to follow Plos guidelines.

Please review our guidelines at <https://journals.plos.org/plosone/s/materials-and-software-sharing#loc-sharing-code> and ensure that your code is shared in a way that follows best practice and facilitates reproducibility and reuse.

We have reviewed the policy and believe our code is in compliance. It is uploaded to Zenodo with the provided DOI ready to be made public on acceptance.

Please provide an amended statement that declares *all* the funding or sources of support (whether external or internal to your organization) received during this study, as detailed online in our guide for authors at <http://journals.plos.org/plosone/s/submit-now>.  Please also include the statement “There was no additional external funding received for this study.” in your updated Funding Statement.

Please include your amended Funding Statement within your cover letter. We will change the online submission form on your behalf.

All funding sources are declared, and the final line has been included. The funding statement is now:

Swedish surveys were funded by the Swedish Environmental Protection Agency, and the Swedish Agency for Marine and Water Management. Danish surveys were funded by the Danish Environmental Protection Agency. DC thanks the Wild Animal Initiative Fellowship, grant number F-2023-00005, and the Swedish Environmental Protection Agency through the Environmental Research Fund and the Swedish Research Council Formas, grant number 2024-00147. KCH was supported by the European Union MARHAB, grant no. 101135307, and the Swedish Research Council Formas, grant no. 2023-00502. There was no additional external funding received for this study.

When completing the data availability statement of the submission form, you indicated that you will make your data available on acceptance. We strongly recommend all authors decide on a data sharing plan before acceptance, as the process can be lengthy and hold up publication timelines.

All data is uploaded to Zenodo with the provided DOI ready to be released on acceptance.

Please include a separate caption for each figure in your manuscript.

Separate captions are included for each figure at the relevant point in the text.

We note that Figure 1 in your submission contain [map/satellite] images which may be copyrighted. All PLOS content is published under the Creative Commons Attribution License (CC BY 4.0), which means that the manuscript, images, and Supporting Information files will be freely available online, and any third party is permitted to access, download, copy, distribute, and use these materials in any way, even commercially, with proper attribution. For these reasons, we cannot publish previously copyrighted maps or satellite images created using proprietary data, such as Google software (Google Maps, Street View, and Earth). For more information, see our copyright guidelines: <http://journals.plos.org/plosone/s/licenses-and-copyright>.

We require you to either (1) present written permission from the copyright holder to publish these figures specifically under the CC BY 4.0 license, or (2) remove the figures from your submission.

As recommended in email correspondence with the editors, we have replaced the administrative boundaries with copywrite free polygons sourced from sourced from Natural Earth.

<https://www.naturalearthdata.com/about/terms-of-use/>

“All versions of Natural Earth raster + vector map data found on this website are in the public domain. You may use the maps in any manner, including modifying the content and design, electronic dissemination, and offset printing. The primary authors, Tom Patterson and Nathaniel Vaughn Kelso, and all other contributors renounce all financial claim to the maps and invites you to use them for personal, educational, and commercial purposes.

No permission is needed to use Natural Earth. Crediting the authors is unnecessary.”

We have updated the Figure 1 legend accordingly.

## Responses to Reviewer #1

The results indicate an annual decline of 408 individuals in the Kattegat-Skagerrak, but the confidence interval is relatively wide (95% CI: [67, -882]). While this reflects uncertainty in the estimates, a brief discussion of the implications of this variability in trend estimates would strengthen the interpretation.

Some reorganization of Introduction might be helpful to streamline the manuscript. It is recommended to check the text for consistency of terminology used and avoid repetitive expansion of acronyms.

The suggested revisions provided in the text primarily focus on minor clarifications and additional context to further strengthen the paper.

The authors would like to thank the reviewer for their time and insightful comments on our manuscript. In line with their suggestions, we have now made updates, in particular to the introduction, methods, and discussion, to clarify the points raised.

We agree that a more detailed discussion of uncertainty will be of value to our readers. As well as updating the methods to explain steps taken to minimize the impact of environmental variability on survey days (L119-121), we have added a section in the discussion addressing these points (L269-276).

We have shortened our description of drivers of harbour seal growth/decline in the introduction and instead address these points in detail in the discussion.

We have also worked to standardise our terminology and now expand acronyms on their first mention in the entire text only rather than per section.

### Specific comments

Title: and Danish Straits?

Here and elsewhere, we agree the inclusion of Danish Straits may not be helpful to readers not familiar with the region. Additionally, we were did not include data on some parts of the Danish straits as discussed L247-255.

In the title, we now focus only on our major finding of the observed changes in the Kattegat-Skagerrak and elsewhere make more reference to the S.W. Baltic as an inclusion in the analysis.

L28 sub-regions or subregions - check for consistency.

This has been amended to subregion in all cases thank you.

L35 Here and in the main text point out when the shift from increasing trend to a declinng occured and try to provide a more focused discussion as to why the change ocured (i.e., what could change around 2017?).

At present, we cannot point to a specific change in 2017 which is likely to have led to the shift but instead believe it is likely a combination of factors acting over a longer time period. We have updated the final sentence in the abstract to address this (L36-40).

We have now also made substantial changes to the discussion to more explicitly address the changes which we believe are likely to have led to declining counts (L295-359).

L44 and Danish Straits?

Now omitted here and elsewhere.

L58 Do you mean that there was a period of growth after the first PDV outbreak? Need to clarify this passage as below it is stated that after the second outbreak the growth was only 5-6.5%

We agree this paragraph was unclear. The 1988 outbreak was followed by growth at above 10% while the 2002 outbreak was followed by lower growth at 5-6.5%. We have rewritten L58-62 to improve the clarity.

L75 Lines 79-109: I would suggest condensing these paragraphs and leaving a major portion of information provided here for the discussion.

As suggested, we have compressed these paragraphs into a few short sentences (L76-78) and now address these potential drivers of change in the discussion (L295-359).

L81 However, grey seals.

Suggestion accepted thank you.

L108 are impacted by..?

Suggestion accepted thank you.

L115 You keep leaving the Danish Straits out - I am not sure whether that's intentional or not because the results contain SW Baltic Sea haulouts so it seems that they should be included in the objectives here and elsewhere.

Rather than referring to the Danish Straits, we now refer to the S.W. Baltic explicitly here and elsewhere to more accurately represent our survey area. We focus more on the Kattegat-Skagerrak, with the inclusion of the S.W. Baltic as a comparison.

L118 perhaps specify that by environmental degradation you mean human-caused changes, assuming that is the case?

We agree this should be highlighted at this point. We have amended the sentence to “This work presents a valuable case study of potential growth limitation for a marine mammal population and an opportunity to begin to disentangle the influence of natural processes from human driven environmental change.”

L124 PDV - was already explained in the Introduction

This has been amended thank you.

L127 However, for management purposes...

Suggestion accepted thank you.

L132 Was it a single survey each year on a single day? As you mention in the discussion, the weather conditions as well as time of the day and a day of the year can have impact on the proportion of seasl available for counts. Perhaps provide a short description here indication whether the survey conditions were similar between years.

Each survey for the different sea regions took place on a single day. We agree additional details on the methodology are helpful here. At your suggestion we have more explicitly indicated the number of survey days (L125-126 & 129-131), timing (L119- 121 and 128-129), and weather conditions (L20-121). We have also reorganised the section (L104-131) in a way that we believe will make the methodology clearer to readers.

L153 could you elaborate why the mean counts were used as opposed to max counts?

This has been the subject of an extensive previous analysis (Tielmann et al. 2010) which we now discuss in text (L137-139).

Figure 1 legend: 1-8 (probably OK to use numbers for subregions)

Suggestion accepted here and throughout the text thank you.

L162 growth rate

Suggestion accepted thank you.

L168 ,

Suggestion accepted thank you.

L169 w

Suggestion accepted thank you.

L170 :

Suggestion accepted thank you.

L180 :

Suggestion accepted thank you.

L183 t

Suggestion accepted thank you.

L197 CI

Suggestion accepted thank you.

L203 perhaps paraphrase to make it clear that only once they were srubeyed in 2004, but were surveyed multiple times in all other years. Otherwise it sounds like they were only surveyed in 2004.

We agree and have updated to the text to clarify this point (L185-187).

L214 ,

Suggestion accepted thank you.

L218 Is it necessary to provide full abbreviation in each caption? GAM has already been explained in text

This has been amended in all captions thank you.

Figure 2:	Were capitalized in the text above - need to check for consistency. Also, perhaps	just use GAM elsewhere since it was already explained earlier.

This has been amended in all captions thank you.

CI

Suggestion accepted thank you.

In

Suggestion accepted thank you.

L228 Not sure why the authors have lower and upper bounds of CIs in wrong order, here and elsewhere?

This was a typo and has been corrected in all cases to [lower CI, upper CI].

L231 was it supposed to be 9, 99?

Yes – corrected here and elsewhere.

L234 Add summary information here on what you found

Summary included thank you (L216-218).

L239 This term seems to be used interchangebly with the Danish Straights which is confusing.

Here and throughout the text, we have now removed reference to the Danish Straits in favour of explicitly referring to the S.W. Baltic population.

L249 Perhaps compare to 1990s-2000s estimates?

We believe it is preferable to compare the raw counts from the 1990s-2000s to present counts (L224-225) to avoid assumptions of the fraction of the population hauled out. There is a good deal of uncertainty around the proportion of the population observed during the moult. In this last section (L228-232), it was necessary to refer to estimates of population size as there were no counts in the 1890s. We have aimed to make the underlying uncertainty clear with the phrasing “Although they should be considered as coarse estimates only”.

L253 leading up to hunting ban?

Suggestion accepted thank you.

L263 Worth mentioning somewhere (in the Methods? or here) whether the surveys withing each region were conducted during one day or multiple days? that could help justify lack of migration between sites during each particular survey.

These details have been included in the methods thank you (L124-125).

L273 those colonies might become...

Suggestion accepted thank you.

L290 Need to explain this. Seals spend less time on the haulouts during the molt? molting season is more extended? or they haulout during a different part of the day? Or haulout elsewhere?

This section has been rewritten to more clearly explain the possibility that competition for haul out sites and behavioural changes (more time foraging) could lead to seals spending less time on land (L277-287).

L304 become

Suggestion accepted thank you.

L304 I don't believe it was mentioned in the results. Would be interesting to see what the value is.

These values are presented in table S2. We now also give them at this point in the text (L339).

L329 I would be caution stating this - not all mass mortality events result in subsequest exponential growths and recovery.

This is true and we did not intend to suggest this was the case – we have replaced “will” with “are expected to” and added the clause “if conditions remain favourable” (L350-352).

## Responses to Reviewer #2

This represents a nice summary of harbor seal trends in a lesser well represented area of the world. However, I felt that the explanation of statistical methods needed more detail, specifically in terms of accounting for changes in survey effort over time, and what models were being compared with AIC.

We would like to thank the reviewer for their time in reviewing our manuscript and highlighting areas in which it can be improved. In line with their suggestions, we have made substantial changes to the methods and results section. In particular, we now provide more detail on survey conditions. AIC was used as a tool for model comparison in the parametric modelling only. It was not used for evaluating the GAM fit in this study. To avoid any confusion, we have removed references to AIC made in relation to the GAMs.

### Specific comments

L28 This clause is a bit confusing

We have removed this clause.

L33 an "annual decline" *in* a specific year is a bit confusing. If it's an annual decline, I'd prefer to see a start and end date included. Annual from 2003 to 2023? I would also be inclined to calculate the percent change (rather than 408 individuals), and calculate the percent change from the previous year rather than a percent of the average counts in 2023? Perhaps I'm misunderstanding.

This is the direct outcome of the derivative of the GAM as per Figure 2B, rather than being an average rate of change across the entire period (which given the mix of positive and negative growth would likely be close to 0), it is the final prediction. We wanted to avoid the subjective selection of a time period for which to calculate an average rate of change. Note that the GAM is fit to the entire series meaning this final value is not based on change between 2022 and 2023 only but all data up to that point.

To avoid confusion, we have included more details on the method in the abstract (L28) and now use the phrasing “annual decline ... at the end of the survey period” (L30). We also now estimate a final rate of change (L31 and L34). We keep the number given in counted seals along with its confidence interval as it is the direct GAM model output.

L41 "Health" could be perceived as a value-laden word, or at least fairly subjective.

We have replaced health with “state” here and in the conclusion.

L43 This is the only mention of climate change in the introduction

We have removed the reference to climate change.

L46 governing -> affecting

Suggestion accepted thank you.

L51 the -> a

Suggestion accepted thank you.

L66 If 12% is the max, I wouldn't call these "low". Seems like strong growth for a pinniped to me

This section has been rewritten (L65-66) for clarity and now says “lower” rather than low.

L82 and L84 orca -> orca whales

We have switched to “killer whale” to match the citation we use.

L95 was -> has

This refers to management in 2024 and so we have kept it in the past tense.

L115 determine -> estimate

Suggestion accepted thank you.

L136 Perhaps there are correction factors or more details about the counting process here? Is it single-counted? Or double counted and reconciled between two analysts? What about the timing of surveys relative to daily seal activity and accounting for individuals not hauled out? Is that consistent from year to year or area to area?

L154 Similar to the above comment, perhaps a bit more detail about survey effort and consistency over time and space would be useful to help readers understand how uncertainty in survey timing/effort was treated in the estimation process.

Details on double counting (L132-134) as well as standardisation of survey conditions have now been included (L119-121).

L183 The -> the

Corrected thank you.

L187 Did you compare these rates to average annual changes in population counts? I'm not sure how this differs from simply using lambda = Nt+1/Nt.....

I'm also curious how sensitive these growth rate are to the choice of knots, particularly how different they are from growth rates calculated from counts as noted above.

We did not calculate annual changes in population count in isolation. We do not believe these would be particularly informative give the large degree of annual variation present in surveys of this kind (Teilmann et al., 2010; White, 2019). They would require some form of model fitting to determine long term trends – bringing us back to the need for a GAM. Parametric modelling (the fitting of a lambda) assumes a steady state environment and does not allow for population decline after a period of growth. The non-parametric modelling (and its first derivative) describes count size/change in count without underlying assumptions which would mask a decline. We have updated the methods (L157-164) and discussion (334-346) to make this purpose of the GAMs clearer.

Regarding knot selection, we did not impose a strict number of knots on the model (L175). Number of knots is not a parameter fed to the gam() function. Instead, you feed it a value “k” which specifies a starting number of basis functions (with each function having an inflection point). The default starting k is 10. This does not mean the final model will have ten basis functions, instead, during the fitting the least number of basis functions will be used to describe the data.

When fitting a gam, it is common practice to either specify a number of starting basis functions based on visual examination of the data or start with an unspecified number (which is really starting with 10) (Wood, 2017). From Wood (2017) “...exact choice of k is not generally critical: it should be chosen to be large enough that you are reasonably sure of having enough degrees of freedom to represent the underlying ‘truth’ reasonably well, but small enough to maintain reasonable computational efficiency.” To examine this, I ran the model fitting for the Kattegat-Skagerrak for a **specified** range of k values from 1 to 9. Beyond 4, the model is over fit with a number of inflection points which are likely the result discussed variability in the surveys (L269-276). 4 basis functions also gives the previously described rates implying that an unspecified number of basis equation results in four final functions. This is not reported in the gam() output. To clarify, I now manually set starting k to 4 and explain its selection in the text (L178). For your interest, I show the outcome of specified k selection below. In all cases there is a decline. I do not believe showing this is necessary in the text as we can reference the common practice as described in Wood (2017).

Teilmann, J., Rigét, F., and Harkonen, T. (2010). Optimizing survey design for Scandinavian harbour seals: population trend as an ecological quality element. *ICES Journal of Marine Science* 67, 952–958. doi: 10.1093/icesjms/fsq001

White, E. R. (2019). Minimum Time Required to Detect Population Trends: The Need for Long-Term Monitoring Programs. *BioScience* 69, 40–46. doi: 10.1093/biosci/biy144

Wood, S. N. (2017). *Generalized Additive Models: An Introduction with R*., 2nd Edn. Chapman and Hall/CRC. doi: 10.1201/9781315370279

L192 From the methods above, it isn't clear what models are being compared using AIC. Is it comparing Eq 1 and Eq 2? I think care is needed when comparing AIC values between linear/fixed effects models and gams. I think more detail about fixed vs. random effects structures here is appropriate if AIC model comparison is used.

We do not compare the parametric and nonparametric models based on AIC – only exponential vs. logistic parametric models. We have updated this section to make this fact clear (L175-177).

L205 Speaks to my above comment - unclear how counts can be summed or averaged if survey effort isn't necessarily consistent over space and time? Perhaps it is fine, but it should be spelled out a bit more clearly.

We have updated this section (L185-187) as well as the methods (L119-131) to make it clear that these subregions are not included in the analysis of trends.

L210 If AIC and R-sq are noted below, it should also be noted here.

AIC has been removed for the GAMs, R^2 are now reported in all cases.

L215 It might be more meaningful to compare AIC's across GAMs from a null model to one that includes year/region and interaction - that seems like the more traditional use and reporting of AIC values, right?

Table 2 AIC values in the table?

We do not intend that GAM models be compared based on AIC – it is likely that the inclusion of smaller geographic breakdowns will enable better fitting models. Rather the purpose of summarising the trends at these different levels is to explore if there is any particular region driving the trends. We have removed the reference to AIC for GAMs to simplify the manuscript.

L227 Report the time frame

This is the final value of the first derivative of the GAM (as seen in figure 2B). As discussed for the abstract it is not for a specific time frame but rather based on the entire series. We have added a sentence to the text to clarify the meaning of these rates (L206-209).

L231 This doesn't seem right based on the mean of 54; 9 to 99?

Corrected thank you – the intervals were in the wrong order.

L289 This should be addressed in the methods relative to survey effort/timing

More details on survey effort and timing are now provided (L119-131).

L316 Are there prey survey data or estimates that could have been included in GAMs as covariates?

This would be an interesting analysis, unfortunately harbour seals in the region regularly consume more than 30 prey species, many of which are non-commercial and therefore poorly studied. Our understanding of how seal diet shifts with prey abundance isn’t sufficient to do this analysis at this time. We do now recommend a more in-depth analysis of dietary change which could incorporate such an analysis on L305-309.

L350 Doesn't seem as though evidence has been provided to make this conclusion

We have updated this section to be more cautious. We are careful here to state that it is “likely” rather than a fact that changes are driven by nutrient limitation and provide the caveat that behavioural changes may have an impact L367-369. We believe this conclusion is justified given well documented changes in the ecosystem explored in the introduction (L78-91) and the exploration of nutrient limitation (and elimination of other common causes of harbour seal decline) in the discussion (L295-333). While we cannot provide a definitive answer as to the cause of reduced growth rates at this time, we believe the multiple lines of evidence we present supports the conclusion that there are real shifts in seal abundance occurring.
